# The Obesity Paradox in Chronic Heart Disease and Chronic Obstructive Pulmonary Disease

**DOI:** 10.7759/cureus.25674

**Published:** 2022-06-05

**Authors:** Suganya Giri Ravindran, Debistuti Saha, Iffat Iqbal, Sharan Jhaveri, Chaithanya Avanthika, Mridula Sree Naagendran, Lakshmi Deepak Bethineedi, Tony Santhosh

**Affiliations:** 1 Internal Medicine, California Institute of Behavioral Neurosciences & Psychology, Fairfield, USA; 2 Medicine, Regional Institute of Medical Sciences, Imphal, IND; 3 Internal Medicine, Dow University of Health Sciences, Civil Hospital Karachi, Karachi, PAK; 4 Internal Medicine, Smt. Nathiba Hargovandas Lakhmichand Municipal Medical College, Ahmedabad, IND; 5 Medicine and Surgery, Karnataka Institute of Medical Sciences, Hubli, IND; 6 Medicine, PSG Institute of Medical Sciences and Research, Coimbatore, IND; 7 General Medicine, Andhra Medical College, Visakhapatnam, IND; 8 Medicine, Dr. Somervell Memorial CSI Medical College, Thiruvananthapuram, IND

**Keywords:** cardiovascular disease, chronic obstructive pulmonary disease, chronic heart failure, paradox, obesity

## Abstract

Obesity in recent years has become an epidemic. A high body mass index (BMI) is one of today's most crucial population health indicators. BMI does not directly quantify body fat but correlates well with easier body fat measurements. Like smoking, obesity impacts multiple organ systems and is a major modifiable risk factor for countless diseases. Despite this, reports have emerged that obesity positively impacts the prognosis of patients with chronic illnesses such as chronic heart failure (CHF) and chronic obstructive pulmonary disease (COPD), a phenomenon known as the Obesity Paradox. This article attempts to explain and summarize this phenomenon.

As it stands, two theories explain this paradox. The muscle mass hypothesis states that obese patients are better adapted to tide through acute exacerbations due to increased reserve because of greater muscle mass. The other theory focuses on brown adipose tissue and its anti-inflammatory effects on the body.

We performed a literature review on research articles published in English from 1983 to the present in the following databases - PubMed, Elsevier, and Google Scholar. The following search strings and Medical Subject Headings (MeSH) terms were used: "Obesity," "Heart Failure," "COPD," and "Cardio-Respiratory Fitness." In this review, we looked at the obesity paradox in Heart Failure and COPD. We summarized the current literature on the Obesity Paradox and reviewed its relationship with Cardio-Respiratory Fitness.

## Introduction and background

Over recent years, there has been a tremendous increase in obesity in both men and women globally [1]. In the International Classification of Disease (ICD), obesity is listed as a chronic disease (ICD-9, E 66.9). It is associated with numerous comorbidities and increased mortality [2,3]. Particularly, obesity has several harmful effects on cardiovascular physiology and function, serving as a significant risk factor for cardiovascular diseases (CVD) such as heart failure (HF) and sudden cardiac death [2,4]. Furthermore, obesity alters the pulmonary function and has been linked to various respiratory diseases such as chronic obstructive pulmonary disease (COPD) and obstructive sleep apnea [5].

Despite these negative consequences, some studies have found that obesity improves the prognosis of chronic illnesses, including chronic heart failure (CHF) and COPD [6-9], a phenomenon known as the "obesity paradox." These findings demonstrated that overweight and obese people with established diseases reaped the most significant benefits compared to the general population (average weight and underweight). It is found that noncommunicable disease-related disability and death, such as CHF and COPD, are on the rise [10,11]. Thus, gathering evidence on the obesity paradox is crucial to understand this slightly unusual and unexpected phenomenon better.

Recent studies have shown that people with COPD who are overweight or have class I obesity had better outcomes with fewer exacerbations. Still, patients with a lower body mass index (BMI) have a high mortality rate [11-14,15]. Moreover, overweight and obese patients with low forced expiratory volume 1 (FEV1) have a favorable prognosis, implying a better outcome in severe disease [7,16]. Aside from that, a growing body of evidence suggests that overweight and obese people with intact cardiorespiratory fitness (CRF) have more favorable impacts on CHF than lean people or who have a normal BMI [17-19]. CRF has been identified as an important predictor of HF survival [20], as assessed by the maximum volume of oxygen uptake (VO2 max) that a person can use during exercise or the ratio of minute ventilation to carbon-di-oxide output (VE/VCO2) (amount of gas inhaled and exhaled per minute/amount of CO2 exhaled per minute from lungs).

Surprisingly, many phenotypes of obesity have been identified, including obesity based on BMI, sarcopenic obesity (progressive loss of skeletal mass with an increase in fat mass due to ageing), metabolically normal/abnormal obesity, and obesity based on malnutrition and obesity with high or low CRF. These traits influence HF morbidity, mortality, and treatment results [21].

Gruberg et al. coined the term "obesity paradox" in 2002 [22]. Following this, more research on the obesity paradox in various conditions has been conducted. This review's primary goal is to explain the obesity pandemic and to analyze the biology and prevalence of the obesity paradox. We have reviewed relevant literature on the obesity paradox's impact on CHF and COPD, including three randomized controlled trials (RCTs) and over a dozen cohort and case-control studies.

## Review

The obesity epidemic and pathophysiology

The Obesity Epidemic

Obesity is defined as an abnormal accumulation or distribution of fat in the body that has negative impacts on health [23]. Obesity has reached pandemic proportions around the world [24-26]. The world's obese population is estimated to be 250 million people, with 300 million expected by 2025 [24]. Obesity prevalence has risen globally since 1980 and has continued to rise in individuals of all ages and genders, regardless of socioeconomic status, geography, or ethnicity [27].

A high body mass index (BMI) is one of today's most medical health issues [28]. BMI was one of the top five risk factors in attributable deaths and disability-adjusted life-years in the Global Burden of Disease Study in 2017, which evaluated 84 other risk factors [29].

Obesity has joined the ranks of chronic diseases and has superseded malnutrition and infectious diseases as the leading causes of death among the general public [30]. Understanding energy balance is essential to understand how the obesity pandemic originated and weight gain occurs when there is a positive energy balance [31].

At present, the established way to categorize obesity is by BMI. BMI = weight (kg)/[height (m)^2^]. The BMI and the considered weight range are depicted in Table 1 [32].

**Table 1:** Body Mass Index (BMI) and the considered weight range

BMI	Considered
BMI less than 18.5	Underweight range
BMI is 18.5 to <25	Healthy weight range
BMI is 25.0 to <30	Overweight range
BMI is 30.0 or higher	Obesity range

Obesity is subdivided into categories which are depicted in Table 2 [32].

**Table 2:** Obesity subdivided into categories by Body Mass Index (BMI)

Categories		BMI
Class 1		BMI of 30 to < 35
Class 2		BMI of 35 to < 40
Class 3		BMI of 40 or higher. Class 3 obesity is categorized as "severe" obesity

BMI does not directly quantify body fat, but it is well correlated with other ways of measuring body fat measurements such as skinfold thickness measurements, bioelectrical impedance, underwater weighing, and dual-energy X-ray absorptiometry (DXA), and other approaches [33-35]. According to the Hales et al. National Health and Nutrition Examination Survey, the prevalence of age-adjusted obesity in adults in the United States was 42.4% [36]. In addition to that, the age-adjusted prevalence of extreme obesity was 9.2%. Severe obesity was higher in females, but the prevalence of obesity was unaffected by age or gender. Adults aged 40 to 59 years had the highest incidence of severe obesity.

Obesity and Chronic Heart Failure (CHF)

Obesity has been associated with diabetes, hypertension, chronic heart failure (CHF), dyslipidemia, gallbladder disease, and some malignancies including kidney, breast and oesophageal, etc. [37-39]. Excess weight is important, but so is the pattern of fat distribution, as it determines the threat in many of these diseases. Gluteofemoral or lower-body fat distribution patterns are more common in women than in men, whereas abdominal or upper-body obesity is more common in men. Women, on the other hand, are more likely than males to accumulate abdominal and upper-body fat when they gain weight [40]. A pattern of upper-body fat distribution has been linked to a higher risk of diabetes and CHF in numerous prospective longitudinal studies [41-46]. In addition, multiple cross-sectional studies have established a correlation between abdominal fat distribution and metabolic abnormalities including atherogenic profile, high fibrinogen levels, hypertension, insulin resistance, hyperinsulinemia, glucose intolerance, arthritis, menstrual irregularities, and gallbladder disease [47-49]. In the United States (US), the annual health expenses for a single obese person were estimated to be US$1,901 in 2014, equating to US$149.4 billion at the national level [50].

Obesity is a potent risk factor for CHF. However, the relative association between obesity and CHF is incongruous and shows a U-shaped association [51]. Surprisingly, several retrospective and prospective studies showed that obesity has a beneficial role in CHF patients compared to CHF patients with normal BMI [9,52,53]. This phenomenon is termed the ‘Obesity Paradox’. More data reveals this condition to be found in conditions like congenital heart disease (CHD), hypertension, atrial fibrillation, pulmonary hypertension, and respiratory distress [7,54,55].

Obesity and Chronic Obstructive Pulmonary Disease (COPD)

Several theories have been stated to explain the phenomenon ‘obesity paradox’ observed in chronic obstructive pulmonary disease (COPD) [56]. The improved prognosis of COPD patients who are on a long-term hospital treatment is explained through the muscle mass hypothesis [57,58]. During acute exacerbations, obese patients are better adapted due to increased reserve because of greater muscle mass. Moreover, respiratory failure is one of the major causes of death in an acute setting. This theory of loss of muscle mass can also explain the poor prognosis of COPD patients since patients who are better tolerant to muscle mass wasting have a higher chance of survival [59]. Based on this hypothesis, metabolically healthy overweight and obese patients who have higher muscle mass (lean muscle mass) have improved chances of recovery than the non-obese population [9].

The second theory is based on the consequences of systemic inflammation [9]. COPD is associated with elevated pro-inflammatory cytokine levels and the fat composition of the body plays a major role in determining the prognosis. The adipose tissue in the body is composed primarily of brown adipose tissue and white adipose tissue. Characteristics of brown adipose tissue behave like the lean mass of a body in that it reduces the level of lipopolysaccharides in the body which stimulates the pro-inflammatory cytokine and thereby effectively reduces the level of systemic inflammation [60]. The white adipose tissue functions exactly the opposite way and has harmful effects on the body. Moreover, conversion of white adipose tissue to brown occurs in some obese individuals [61].

Certain benefits provided by the fat-free mass in the obese population are protection from oxidative and inflammatory stress and also better tolerance to weight loss and associated fatigue in patients with chronic diseases involving the cardiovascular or respiratory system [62]. Another theory of reverse causality tries to explain this condition by stating that the non-obese population, due to a history of prior health conditions, acute or chronic, has suffered a loss of weight leading to low BMI. Their poor health condition acts as a potential risk factor for poor prognosis against COPD, and thus when compared to the comparatively healthy obese population, the latter shows a better prognosis [63].

Confounding Variables

Several confounding factors like gender, age, socioeconomic status, and therapeutic strategies are not taken into consideration in the studies of the paradoxical effect of obesity on the prognosis of CHF [64]. Furthermore, the use of BMI as the standard tool of measurement does not take into consideration the variation in body composition. Rather, cardiorespiratory fitness and methods like abdomen to hip ratio are more accurate tools of measurement [65].

Obesity paradox in CHF

Obesity is a substantial risk factor for heart failure (HF) in both patients with reduced ejection fraction (HFrEF) and those with preserved ejection fraction (HFpEF) [66]. BMI has been seen to be directly proportional to the incidence of HF [67,68]. However, in HF, the phenomenon ‘obesity paradox’ has been observed prompting several studies to explain this correlation [67].

Obese patients appear to have lower mortality than people with average BMI after the onset of HF [69]. Cicoira et al. compared the association of obesity with CHF mortality finding that the mortality rate was 27.2% in patients with BMI less than 22 kg/m^2^ (underweight), 21.7% in patients with BMI 22-24.9 kg/m^2^ (average weight), 16.5% in patients with BMI more than 30 kg/m^2^ (obese) [70]. Moreover, subsequent studies demonstrated that obesity has a favorable influence on CHF mortality; survival rates are better in overweight and obese patients than in normal-weight subjects [7,71]. Hospitalization rates are lower in overweight individuals than in people with normal or underweight [72]. Surprisingly, obesity, in both HFrEF and HFpEF, was found to reduce mortality in HF regardless of the ejection fraction [73].

In addition to BMI, other factors played a part in the obesity paradox, as previously indicated. To better understand these variables, when BMI is divided into two distinct aspects, body lean mass (LM) and fat mass (FM), a greater LM and low FM are associated with a better prognosis in chronic heart failure (CHF) [74]. In addition, there is evidence that fitness impacts BMI and mortality [4]. Following this, other coronary artery disease and HF studies underline the importance of cardiorespiratory fitness (CRF) [75,76]. According to these researches, the higher the CRF, the better the prognosis [77]. Also, Carbone et al. revealed that obese subjects usually have an excess of LM (as compared to the mean LM for their, gender and height), which partly explains the obesity paradox results in a better outcome when combined with better CRF. Thus, a higher CRF is one of the factors contributing to favorable long-term outcomes for CHF patients [78].

HF is a long-term condition that increases catabolism in an individual. As a result, it causes a condition known as cardiac cachexia (weight loss of 7.5 percent of prior average weight in individuals with CHF for at least six months without evidence of other primary cachectic disorders (e.g., cancer, thyroid disease, or severe liver disease)) [79]. Cachexia seems to have a marked effect on the prognosis of HF patients; in an 18-month follow-up, 50% of those with cardiac cachexia posed a high mortality rate. Therefore, cardiac cachexia is predictive of poor outcomes in CHF irrespective of age and left ventricular ejection fraction (LVEF) [80].

An interesting study, the CHARM (Candesartan in Heart Failure: Assessment of Reduction in Mortality and Morbidity), emphasized the insignificance of weight loss advice to CHF patients as it has no remarkable effect on their prognosis [81]. Weight loss is a poor prognostic factor for HFrEF in normal/underweight non-diabetic patients having an increased risk of one-year mortality, excluding obese and diabetic patients [82]. Furthermore, a Cohort study contrasted obese and non-obese hospitalized patients. Obese adults with CHF had a mortality rate of 1.8%, while non-obese patients had 3.1%. Also, 19.4% of obese patients had been readmitted within 30 days compared to 20.9% of non-obese patients [83].

Obesity paradox in COPD

The obesity paradox in COPD has not been completely understood. Because the obesity paradox appears to have a role in influencing outcomes in people with a variety of chronic conditions, it is only a proportional assumption to expect comparable findings in people with COPD. COPD patients with a higher BMI may experience more dyspnea and activity limitations but are found to experience a comparatively favorable prognosis compared to patients with normal BMI [84].

Patients with COPD are more likely to become obese as a result of reduced physical activity and long-term use of systemic glucocorticoids [85]. Fat-free mass (FFM), particularly muscle mass, is lost as a result of systemic inflammation, which is accompanied by an increase in fat mass. In moderate to severe COPD patients, loss of FFM causes muscle weakness and a decrease in exercise capacity, making the FFM index a potentially stronger predictive indicator than BMI in COPD [86]. Weight loss, in addition to FFM, has been proven to affect the prognosis of COPD patients [87].

Obese patients with severe COPD seemed to have the lowest mortality rate, according to Landbo et al. [16]. Similar findings were found in a population-based study by Jee et al., who found that people with a higher BMI had a decreased risk of dying from pulmonary conditions [51]. Underweight patients had increased mortality risks than those with a normal BMI, according to Cao et al. [6]. Górecka et al., in a study focused on observing the effects of long-term oxygen therapy on survival in patients with COPD, discovered that patients with higher BMIs (>25 kg/m^2^) had significantly better survival rates than their lean equivalents (<20 kg/m^2^) with a p-value of 0.035. The Hazard ratio, estimated by Cox Regression using BMI as an independent variable, was found to be 0.942 (95% CI=0.905 to 0.996). These results were further adjusted for FEV1, and BMI was found to be a significant predictor of survival independent of FEV1 [88].

Blum et al. [89] discovered that patients suffering from COPD and peripheral artery disease (PAD) had reduced abdominal obesity and lung disease deterioration. The mortality was greater in individuals without PAD and similar in symptomatic and asymptomatic patients with PAD because of decreased endothelial function. These observations of studies support the existence of the obesity paradox in COPD patients.

Wu et al. conducted a cohort study to determine the obesity paradox role in smoking-induced COPD and non-smoking-induced COPD (other causes). The study has shown that mortality rate decline was present only in smoking-induced COPD patients and not seen in non-smoking-induced COPD patients. Thus, the paradox of obesity was believed to be limited to only smoking-induced COPD [90]. Further studies in pathophysiological variations of these factors can lead to more confirmatory evidence.

The obesity paradox, besides being implied to be supportive for COPD patients, needs to be studied in more extensive detail. Further studies to be conducted might focus on decreasing confounding factors such as age, gender, and other associated factors.

Obesity paradox and CRF

Although an obesity paradox has been seen in mild and moderately obese patients with COPD and HF, it should not be seen as a promotion of obesity in these individuals [91,92]. Even though various studies suggest that weight loss in these patients is associated with an increase in mortality rate, proper guidelines have not been laid regarding weight management as a treatment protocol [2,93]. When discussing the prognosis of these patients with obesity, assessing CRF has been recently highlighted by the scientific statement of the American Heart Association [94]. Research based on body fat and lean mass rather than BMI shows a decrease in mortality after losing body fat [30,95]. Therefore, using BMI as a criterion for treatment is questioned by several researchers [96,97].

Accessory muscles become a major factor in the patient's ability to breathe in COPD, especially during exacerbation and respiratory failure [57]. Studies found that higher physical activity slows the decline of lung function, thereby delaying the progression of COPD [98,99]. CRF improves the respiratory reserve and is thus shown to be a better predictor of outcomes in obese COPD patients [57].

Sabino et al., with their studies on COPD patients, found that obese individuals have higher fat-free mass and thus better exercise capacity and outcome [100]. McAuley and Beavers showed that an unfit man has double the mortality risk as a fit man despite the obesity paradox. Thus, CRF is a better predictor of mortality outcomes than BMI [101]. However, the role of intentional weight loss is still undetermined [100].

Studies on CHD and HF have shown that the obesity paradox exists only in low CRF individuals [77]. A study by Lavie et al. supports this, as they discovered an obesity paradox in patients with peak oxygen consumption of less than 14mlO2/kg/min, but no contradiction was present in patients with good CRF [102]. In a study that evaluates the association of CRF and obesity on mortality, CRF was seen to mitigate the obesity paradox, with mortality being lower in optimum CRF patients [103]. These findings were supported by many other studies, which collectively assume that CRF alters the obesity paradox seen in HF [77,104]. CRF also lowers cardiovascular risk factors like blood pressure and inflammation and increases muscle mass [105].

The relative contribution of fitness and fatness to the prognosis of these patients is still arguable. But recent studies suggest that CRF remains the major contributory factor and it tones down the adverse effects of obesity and other cardiovascular risk factors, diabetes mellitus, and hypertension [106,107]. Therefore, physical activity such as aerobic exercise to maintain CRF and resistance training to improve lean mass should be given more attention [108]. A recent meta-analysis concluded that physical activity has a significant impact on decreasing mortality in CVD [109]. Few studies have assessed the effect of weight loss despite the obesity paradox and suggest weight loss is associated with increased mortality [110,111]. Hence Simonenko concluded that further studies are required to make weight management recommendations after recognizing the beneficial effects of weight loss [112].

## Conclusions

Obesity is a known risk factor for countless diseases, with a high BMI being one of today’s most crucial population health indicators. Yet it appears that the role of intentional weight loss in many health conditions is still undetermined. Moreover, the reduced mortality in obese COPD and HF patients, as seen in multiple studies highlighted in this review demands attention.

This paradox, besides being implied to be supportive for COPD and Heart Failure patients, needs to be studied in extensive detail, with more trials needed to rule out confounders prevalent in previous studies. Cardio-respiratory fitness is a major modifier of this relationship and quantifying its impact can help us better understand this paradox.
